# Genetic predictors of neurocognitive outcomes in survivors of pediatric brain tumors

**DOI:** 10.1007/s11060-023-04472-7

**Published:** 2023-10-25

**Authors:** Sydney T. Grob, Kristen R. Miller, Bridget Sanford, Andrew M. Donson, Kenneth Jones, Andrea M. Griesinger, Vladimir Amani, Nicholas K. Foreman, Arthur Liu, Michael Handler, Todd C. Hankinson, Sarah Milgrom, Jean M. Mulcahy Levy

**Affiliations:** 1 Department of Pediatrics, University of Colorado School of Medicine, Aurora, CO 80045 USA; 2 Morgan Adams Foundation Pediatric Brain Tumor Research Program, Children’s Hospital Colorado, Aurora, USA; 3 Department of Radiation Oncology, University of Colorado Anschutz, Aurora, CO USA; 4 Department of Neurosurgery, Children’s Hospital Colorado, Aurora, CO USA; 5 Department of Pharmacology, University of Colorado School of Medicine, Aurora, CO 80045 USA

**Keywords:** Pediatric brain tumor, Neurocognitive outcome, Single nucleotide polymorphism (SNP)

## Abstract

**Background:**

Neurocognitive deficits are common in pediatric brain tumor survivors. The use of single nucleotide polymorphism (SNP) analysis in DNA repair genes may identify children treated with radiation therapy for brain tumors at increased risk for treatment toxicity and adverse neurocognitive outcomes.

**Materials:**

The Human 660W-Quad v1.0 DNA BeadChip analysis (Illumina) was used to evaluate 1048 SNPs from 59 DNA repair genes in 46 subjects. IQ testing was measured by the Wechsler Intelligence Scale for Children. Linear regression was used to identify the 10 SNPs with the strongest association with IQ scores while adjusting for radiation type.

**Results:**

The low vs high IQ patient cohorts were well matched for time from first treatment to most recent IQ, first treatment age, sex, and treatments received. 5 SNPs on 3 different genes (*CYP29, XRCC1*, and *BRCA1*) and on 3 different chromosomes (10, 19, and 17) had the strongest association with most recent IQ score that was not modified by radiation type. Furthermore, 5 SNPs on 4 different genes (*WRN, NR3C1, ERCC4, RAD51L1*) on 4 different chromosomes (8, 5, 16, 14) had the strongest association with change in IQ independent of radiation type, first IQ, and years between IQ measures.

**Conclusions:**

SNPs offer the potential to predict adverse neurocognitive outcomes in pediatric brain tumor survivors. Our results require validation in a larger patient cohort. Improving the ability to identify children at risk of treatment related neurocognitive deficits could allow for better treatment stratification and early cognitive interventions.

**Supplementary Information:**

The online version contains supplementary material available at 10.1007/s11060-023-04472-7.

## Introduction

An estimated 5,230 children and teens (0–19 years) will be diagnosed with a brain tumor in the United States in 2023 [1], making it the second most common pediatric cancer behind leukemia. As the overall survival of these children has improved from 57 to 74% in the interval from 1975 to 2002 [2] with 5-year survival reaching 75.5% in the time interval between 2013 and 2019 [3] there is increasing concern over the neurocognitive deficits that accompany treatment for brain tumors. In addition to treatment related risk factors such as radiation dose [4], volume and the use of proton radiation in place of photon radiation [5] and surgery [6], several clinical risk factors for neurocognitive deficits have been identified. Some of these include sex [7, 8], younger age at diagnosis [4, 9], and children with hydrocephalus at presentation [10]. Other global considerations for risk of neurocognitive deficits include genetic abnormalities such as neurofibromatosis type-1 [11], socioeconomic status [12] and the level of parental education [13]. More defined biologic markers will make it possible to better predict patients at risk of long-term adverse neurocognitive effects following radiation therapy. This information could allow for better treatment stratification and early cognitive intervention in at-risk individuals.

There is mounting evidence of a link between the ability to repair DNA damage and not only the development of cancer, but also the response to therapy [14]. This is important for pediatric brain tumor patients as standard of care therapy involves DNA damaging agents such as radiation and chemotherapy. We hypothesized that somatic genetic variants in DNA repair genes may be associated with lower IQ scores in children treated for brain tumors. We genotyped 46 children who had been treated for a pediatric brain tumor and assessed whether SNP array profiles were associated with differences in IQ scores.

## Materials and methods

### Patient characteristics

Eligible patients had been previously treated for a brain tumor at Children’s Hospital Colorado and the University of Colorado Denver. Treatment included any combination of surgery, chemotherapy, and photon radiation therapy. We excluded children with known neurocognitive deficits prior to the initial diagnosis of the brain tumor. All children underwent IQ testing with the Wechsler Intelligence Scale for Children (WISC) as part of routine clinical follow-up. Our Institutional Review Board approved this study (COMIRB 08–0985) and patients were prospectively consented according to institutional standards. All patients who had consented to COMIRB 08–0985, had a successful blood sample collection, and had completed a full WISC evaluation with their neurocognitive evaluations before 2016 were included for analysis.

### Laboratory methods

DNA was extracted from patient blood samples using the Qiagen DNAeasy kit, per kit instructions. The DNA was analyzed using a Human 660W-Quad v1.0 DNA analysis BeadChip (Illumina) per the Infinium HD assay protocol as previously published [15]. Data output identified alleles by A and B designations, which were then converted to corresponding nucleotides after statistical analysis for further comparison.

### Statistical analysis

Continuous variables were summarized with mean and standard deviation (SD), if normally distributed. Comparisons by IQ group (< = 90 versus > 90) were performed using two-sample independent t-tests. An IQ of 90 was chosen as it’s the low end of the Wechsler Intelligence Scale for Children (WISC-V) IQ classification for “average” IQ [16]. If not normally distributed, continuous variables were summarized with median and interquartile range (IQR), and a Wilcoxon rank sum test was used to compare across IQ groups. Categorical variables were summarized with frequency and percentage, and comparisons were performed with a Chi-square or Fisher’s exact test.

*Data cleaning* There were 47 patients in the original dataset. This contained serial IQ test results and basic clinical information such as age, sex, diagnosis, radiation, and treatment details. One patient was unable to be linked to the SNP dataset so was removed from the final analysis. There were originally 732 unique SNPS whereby each SNP was categorized into AA versus other (AB, BB, NC), but 118 SNPS only had either all AA or all other so were removed from the final analysis leaving 614 for analysis.

*Most recent IQ outcome* For each of the 614 SNPs, a series of linear regression models were fit. For each SNP, a model with the outcome of most recent IQ, predictors of allele (AA vs. others) and radiation type (CSI vs. focal), and an interaction between allele and radiation type was fit. If the interaction term was significant (p < 0.05), then that model was reported. If not, then the interaction was removed.

*Change in IQ outcome* Only 24 patients who had at least 2 serial IQ measurements were used for analysis. Forty-nine SNPs were not included because they only had a single allele in this sub-population. Using the resulting 565 SNPs, the same series of linear regression models were fit, except each model used the change in IQ (most recent—next recent) as the outcome, and predictors of allele, radiation type, next recent IQ score, and the time between the two IQ measurements. The same decisions were made with the interactions between allele and radiation type.

It was decided a priori that the results would be ranked by the p-value of the allele term (or allele*radiation term, when applicable), and the most significant 10 SNPs would be reported for each outcome (most recent and change in IQ). Analysis was done in R version 4.2.1, and the significance level was set at 0.05.

## Results

Forty-six patients were enrolled in the study and had blood samples obtained for SNP genotyping and at least one IQ test completed. For descriptive purposes, the most recent IQ scores were dichotomized at 90 with 26 (56%) subjects having an IQ less than 90 and 20 (43%) subjects having an IQ greater than 90. The low vs high IQ patient cohorts were well matched for time from first treatment to most recent IQ test (median (IQR): 5.1 (2.7–7.6) vs 3.9 (2.7–4.6) years; p = 0.13), age at first treatment (6.2 (4.3–8.5) vs 6.2 (4–11.2) years; p = 0.71), and sex distribution (65% vs 70% male; p = 0.99). The cohorts differed non-significantly in their primary tumor diagnosis with 54% of patients in the low IQ being diagnosed with medulloblastoma in comparison to ependymoma (25%) and other (25%) being the most common diagnoses in the high IQ group. The primary tumor location for patients in both the low and high IQ groups was the posterior fossa (58% and 45%, respectively); although, the two groups’ tumor locations were significantly different (p = 0.02). The higher IQ group’s second most common location was the suprasellar/hypothalamic (25%) followed by the parietal (10%) and pineal (10%) regions which differed from the low IQ group where the second most common location was the thalamus (15%) and suprasellar/hypothalamic (12%). The groups did not differ in terms of proportion receiving chemotherapy (p = 0.08), radiation type (p = 0.22), CSI dose (p = 1), boost dose (p = 1), focal dose (p = 0.08) or proportion who underwent surgery (p = 0.57). Other patient characteristics and details of treatment can be reviewed in Table 1.

**Table 1:** Demographics and Clinical Characteristics

Characteristic	N	IQ < 90 (n = 26)	IQ > 90 (n = 20)	P-val
Time from first treatment to most recent IQ^†^ *years*	42	5.1 (2.7, 7.6)	3.9 (2.7, 4.6)	0.13
Age at first treatment^†^ *years*	42	6.2 (4.3, 8.5)	6.2 (4, 11.2)	0.71
Most recent IQ	46	73 ± 14.6	101.4 ± 11.4	< 0.0001
*Sex*	46			0.99
Female		9 (35%)	6 (30%)	
Male		17 (65%)	14 (70%)	
*Relapse*	46			1
No		15 (58%)	11 (55%)	
Yes		11 (42%)	9 (45%)	
Diagnosis*	46			0.09
Craniopharyngioma		1 (4%)	3 (15%)	
Ependymoma		2 (8%)	5 (25%)	
Low grade glioma		4 (15%)	2 (10%)	
Medulloblastoma		14 (54%)	4 (20%)	
Non-germinomatous germ cell tumor		0 (0%)	1 (5%)	
Other		5 (19%)	5 (25%)	
Tumor location*	46			0.02
4th ventricle		2 (8%)	0 (0%)	
Other		0 (0%)	1 (5%)	
Parietal		0 (0%)	2 (10%)	
Pineal		0 (0%)	2 (10%)	
Pituitary		0 (0%)	1 (5%)	
Posterior fossa		15 (58%)	9 (45%)	
Suprasellar/Hypothalamic		3 (12%)	5 (25%)	
Temporal		2 (8%)	0 (0%)	
Thalamus		4 (15%)	0 (0%)	
Chemotherapy*	46			0.08
No		3 (12%)	7 (35%)	
Yes		23 (88%)	13 (65%)	
*Radiation type*	46			0.22
Cranial spinal irradiation and focal boost		15 (58%)	7 (35%)	
Focal		11 (42%)	13 (65%)	
CSI dose, categorical*	22			1
< = 24 GY		11 (73%)	5 (71%)	
36 GY		4 (27%)	2 (29%)	
Boost dose, categorical*	22			1
< 20 GY		4 (27%)	2 (29%)	
> = 30 GY		11 (73%)	5 (71%)	
Focal dose	24	57.3 ± 3.2	53.3 ± 6.9	0.08
Surgery*	46			0.57
Biopsy		4 (15%)	4 (20%)	
Gross total resection		6 (23%)	7 (35%)	
Subtotal resection		16 (62%)	9 (45%)	

^†^Skewed outcome: median (interquartile range) and Wilcoxon rank sum test

*Fisher’s Exact Test (due to small cell counts)

Table 2 reports the linear regression results of the 10 SNPs that showed the strongest association between allele (AA vs. other) and most recent IQ result, after adjusting for radiation type. Of the 10 SNPs reported, there was evidence that the association with most recent IQ score for 5 SNPS was not modified by radiation type. These 5 SNPs were located on 3 different genes (*CYP2C9, XRCC1*, and *BRCA1*) on 3 different chromosomes (chromosome 10, 19, and 17). BRCA1 on chromosome 17 had 3 different SNPs whose association with most recent IQ score was not modified by radiation type. Patients with the non-dominant allele in CYP2C9 were associated with a higher IQ compared to those who did not, after adjusting for radiation type (estimated least squares mean (95% CI): 93 (86,99) vs 74 (66,82), respectively). Patients with the dominant allele for *XRCC1* on chromosome 19 had a higher IQ (estimated 90 (84,97) vs 74 (65,84) respectively) after adjusting for radiation type. Lastly, for all three SNPs in *BRCA1* on chromosome 17, those with the dominant allele had a higher IQ compared to those without (estimated 93 (85,101) vs 78 (71,85)) after adjusting for radiation type. There were 5 SNPS in which there was evidence that the type of radiation received modified the association between allele and most recent IQ, and these results are presented in Table 2 where the Interaction Column is equal to “Y”.

**Table 2:** The association between SNP allele (AA vs other) and most recent IQ scores between low (< 90) and high (> 90) IQ groups

SNP	Gene name	Chromosome	pval	N	Interaction?	Least square mean (95% CI) of recent IQ value			
						CSI, AA	Focal, AA	CSI, non-AA	Focal, non-AA
rs2107465	*NBN*	8	0.00052	43	Y	41 (17, 64)	110 (86, 133)	84 (77, 92)	88 (81, 95)
rs12772675	*CYP2C9*	10	0.00056	44	N	74 (66, 82)	74 (66, 82)	93 (86, 99)	93 (86, 99)
rs2735385	*NBN*	8	0.00082	43	Y	40 (17, 64)	103 (83, 122)	84 (77, 92)	88 (81, 95)
rs389480	*BLM*	15	0.00276	43	Y	91 (79, 103)	78 (66, 91)	74 (65, 84)	96 (87, 105)
rs3213403	*XRCC1*	19	0.00609	44	N	90 (84, 97)	90 (84, 97)	74 (65, 84)	74 (65, 84)
rs1374001	*NR3C1*	5	0.00621	43	Y	77 (68, 85)	94 (86, 103)	97 (79, 115)	78 (63, 92)
rs8110090	*TGFB1*	19	0.00696	43	Y	78 (70, 86)	94 (86, 102)	96 (75, 116)	70 (52, 88)
rs16940	*BRCA1*	17	0.00699	44	N	93 (85, 101)	93 (85, 101)	78 (71, 85)	78 (71, 85)
rs16942	*BRCA1*	17	0.00699	44	N	93 (85, 101)	93 (85, 101)	78 (71, 85)	78 (71, 85)
rs1060915	*BRCA1*	17	0.00699	44	N	93 (85, 101)	93 (85, 101)	78 (71, 85)	78 (71, 85)

Table 3 reports the results from linear regression for the 10 SNPs that showed the strongest association between allele and change in IQ, after adjusting for radiation type, first IQ, and years between first and second IQ measures. Of the 10 SNPs, there was evidence that for 5 SNPS the association with change in IQ score was not modified by radiation type, first IQ, or years between first and second IQ measures. These 5 SNPs laid on 4 different genes (*WRN, NR3C1, ERCC4, RAD51L1*) on 4 different chromosomes (chromosome 8, 5, 16, 14, respectively). Patients with the dominant SNP allele had a greater decrease in IQ for SNP rs12677942 on gene *WRN* (estimated decrease in IQ of -31(95% CI − 46,-16) vs -1(− 6,3)), SNP rs2121152 on gene *NR3C1* (− 12(− 18,-6) vs 2(− 3,8)), SNP rs7185124 on *ERCC4* (− 38 (− 59, -17) vs -2 (− 7, 2)), SNP rs17106125 on gene *RAD51L1* (− 38 (− 59, -17) vs -2 (− 7, 2)), and SNP rs7712869 on gene NR3C1 (− 21 (− 34, -9) vs-1 (− 6, 3)). There were also 5 SNPS in which there was evidence that the type of radiation received modified the association between allele and change in IQ, and these results are presented in Table 3 where the Interaction Column is equal to “Y”.

**Table 3:** The association between SNP allele (AA vs other) and change in IQ scores between low (< 90) and high (> 90) IQ groups

SNP	Gene name	Chromosome	pval	N	Interaction?	Least square mean (95% CI) of change in IQ*			
						CSI, AA	Focal, AA	CSI, non-AA	Focal, non-AA
rs9514823	*LIG4*	13	0.00042	19	Y	− 4 (− 12, 5)	− 24 (− 37, − 11)	− 11 (− 18, − 5)	5 (− 1, 11)
rs12677942	*WRN*	8	0.00076	20	N	− 31 (− 46, − 16)	− 31 (− 46, − 16)	− 1 (− 6, 3)	− 1 (− 6, 3)
rs2121152	*NR3C1*	5	0.00210	20	N	− 12 (− 18, − 6)	− 12 (− 18, − 6)	2 (− 3, 8)	2 (− 3, 8)
rs7185124	*ERCC4*	16	0.00294	20	N	− 38 (− 59, − 17)	− 38 (− 59, − 17)	− 2 (− 7, 2)	− 2 (− 7, 2)
rs17106125	*RAD51L1*	14	0.00294	20	N	− 38 (− 59, − 17)	− 38 (− 59, − 17)	− 2 (− 7, 2)	− 2 (− 7, 2)
rs7712869	*NR3C1*	5	0.00555	20	N	− 21 (− 34, − 9)	− 21 (− 34, − 9)	− 1 (− 6, 3)	− 1 (− 6, 3)
rs799917	*BRCA1*	17	0.00592	19	Y	− 1 (− 15, 14)	− 20 (− 36, − 5)	− 10 (− 17, − 3)	5 (− 2, 11)
rs2347869	*ESR1*	6	0.00652	19	Y	− 19 (− 34, − 4)	9 (0, 18)	− 6 (− 13, 1)	− 8 (− 17, 1)
rs726281	*ESR1*	6	0.00665	19	Y	− 16 (− 28, − 4)	9 (0, 18)	− 6 (− 13, 1)	− 8 (− 17, 1)
rs330792	*MSH6*	2	0.00728	19	Y	− 10 (− 17, − 2)	3 (− 3, 10)	− 6 (− 18, 6)	− 32 (− 54, −11)

* Change in IQ = IQ_1 − IQ_2 (most recent − second most recent). So a positive change = increase in IQ over time, negative change = decrease in IQ over time

## Discussion

There has been interest in examining the genetic factors that underlie normal tissue sensitivity to therapy. Numerous groups have examined genetic polymorphisms and radiation toxicity in adults with malignancies of the breast, prostate, head and neck, cervix, endometrium, and lung [17]. One study investigated the potential association between cognitive outcomes and single nucleotide polymorphisms (SNPs) in catechol-o-methyl transferase (*COMT),* brain-derived neurotrophic factor *(BDNF),* and dystrobrevin-binding protein 1 (*DTNBP).* These genes are associated with memory and daily functioning, and all genes are implicated in neurological impairment in adult tumor patients [18]. Other SNPs in the *DIO1* gene, associated with control of thyroid hormone metabolism, were found to have a significant prognostic value in adult glioblastoma patients [19]. Most recently, a study found SNP polymorphisms in genes associated with aging, inflammation, dopamine, myelin cell cycle regulation, and DNA repair may be associated with neurocognitive outcomes in adult CNS tumor patients treated with radiation and chemotherapy [20].

There is a paucity of similar studies in children. In pediatric leukemia, certain SNPs, such as *UGT2B17,* have been correlated with treatment toxicity [21], neurocognitive outcomes [22, 23], and overall mortality. A deletion polymorphism in *UGT2B17* is thought to suppress tumor growth, which could contribute to an overall greater prognosis and reduced chance of relapse [24]. Polymorphisms in the *ACYP2* [25] and *SOD* [26] genes have also been associated with differences in cisplatin-induced ototoxicity. These studies focused primarily on SNPs in genes involved in folate metabolism, drug detoxification DNA repair genes [27].

Evidence regarding the use of genetic profiling in the treatment of brain tumor patients is limited. Previous studies have utilized SNP analysis of the primary tumor to help determine treatment response, but there is little data predicting treatment toxicity. SNP analysis of Glutathione S-transferase [GST] has been studied in relation to neurocognitive toxicity. GST is an enzyme that catalyzes glutathione conjugation of alkylating agents, platinum compounds, and free radicals produced by radiation. A SNP analysis of *GST* in medulloblastoma patients found that the presence of a null genotype was associated with a significant decline in IQ after treatment compared to patients with a no null genotype [28]. Another study found that *GSTP1 105 AG/GG* genotypes were much more likely to experience radiation induced hearing loss. Furthermore, the G allele in combination with high dose radiation was associated with greater risk of treatment-induced toxicity overall [29].

A study of participants in the Childhood Cancer Survivor Study (CCSS) evaluated *GST* and other antioxidant enzyme SNPs to determine if they were associated with neuropsychological impairment. On a Brief Symptoms Inventory-18 questionnaire, patients with a *GST* null genotype reported increased anxiety, depression, and global distress compared to patients with a non-null genotype. But while the CCSS Neurocognitive Questionnaire found poorer functioning in task efficiency and memory by self-report when patients treated for medulloblastoma were compared to sibling controls, there was no difference between genotypes associated with this select set of antioxidant enzymes [30]. A study looking at the association between *COMT* polymorphisms*,* coding for an enzyme used in the metabolism related to control of dopamine levels within the prefrontal cortex and working memory in pediatric brain tumor survivors found that patients with the Met/Val polymorphism variant had a greater working memory performance [31].

More recent research has investigated the association between the three most common polymorphisms in Vitamin D located within the Bsm-1, Fok-1, and Taq-1 regions of the receptor in pediatric brain tumors [32]. The vitamin D receptor binds calcitriol which is involved in several cell processes including cell proliferation, apoptosis, tumorigenesis, cell invasion, and inflammatory response [33]. This study found that the association between polymorphisms in the Vitamin D receptor and cancer development was insignificant, but no research was done correlating identified polymorphisms in Vitamin D to response to treatment [32]. Additional studies look to identify how SNPs may be used in the diagnostics and prognosis of brain tumors [34], but do not correlate these SNPs to toxicity outcomes.

A further study looked to evaluate p53 Arg72Pro polymorphism as an early detector of tumor progression in pediatric astrocytoma and found that having the Arg/Arg72 variant can be used to predict early tumor growth in partially resected astrocytomas. This study went further to suggest that this polymorphism could be used to inform and predict individual response to therapy [35]. The most recent (and to date most comprehensive) analysis [36] was able to longitudinally monitor 241 patients that were treated with CSI and perform a genome wide association study (GWAS) to identify SNP associated with a measured cognitive decline. Their identified variants differed from those discussed through our study but did identify previously identified genes of interest including variants in *GSTP1* and *COMT*. They also identified novel loci related to *PPARD* and *PPARA*. A difference in identified SNP is not unexpected as our study focused specifically at an evaluation of genes associated with DNA damage repair. Collectively, this report as well as our study support the potential importance and clinical usefulness of identifying genetic markers of neurocognitive outcomes in pediatric brain tumor patients.

Our data offers additional insight into the genetic predictors of neurocognitive outcomes in pediatric brain tumor patients and suggests the SNPs identified above in DNA repair genes could be an important tool to assess adverse neurocognitive outcomes in pediatric brain tumor survivors. Genetic polymorphisms are increasingly studied as possible predictors of treatment toxicity. With the exception of the most recent study [37], studies evaluating SNPs as predictors for neuropsychological impairment [28, 30], they have restricted analysis to four or fewer genes at a time and usually focused on genes associated with antioxidant enzymes. Other studies have focused on SNPs associated with the folate pathway. Methotrexate, a folate antagonist, is an important component of leukemia therapy and is a well-described cause of neurocognitive toxicity [38]. In a study of 72 pediatric ALL survivors, genetic polymorphisms in genes involved in folate metabolism were found to correlate with deficits in attention and processing speed [22]. Methotrexate is also used in pediatric brain tumor patients in a selection of radiation sparing (or radiation limiting) protocols such as ACNS0333 and ACNS0334.

Of the 5 most important SNPs in which the interaction with most recent IQ score was not modified by radiation type, the SNPs were found in the *CYP2C9, XRCC1,* and *BRCA1* gene. *CYP2C9* is a cytochrome P450 protein that catalyzes many reactions involved in drug metabolism and synthesis of cholesterol, steroids, and other lipids (www.genecards.org). *XRCC1* is a gene involved in repair of ionizing radiation and alkylating agent induced DNA single-stand breaks (www.genecards.org). *BRCA1* encodes a nuclear phosphoprotein that plays a role in maintenance of genomic stability and secondarily acts as a tumor suppressor (www.genecards.org).

Furthermore, the SNPS on 4 different genes *WRN, NR3C1, ERCC4, RAD51L1* whose interaction with change in IQ score was not modified by type of radiation, first IQ score, or time between first and second IQ score also have unique roles in DNA damage control and regulation. *WRN* encodes for a DNA helicase involved in DNA repair, *NR3C1* a glucocorticoid response gene transcription activator, *ERCC1* in nucleotide excision repair, and *RAD51L1* in homologous recombination and repair (www.genecards.org).

Among the SNPs whose interaction with IQ score (*XRCC1* and *BRCA1*) or change in IQ score (*RAD51L1*) was not modified by radiation type are part of the RAD51 pathway. *RAD51* is the central recombinase protein involved in HR that is vital in the error free repair of DSBs within mammalian cells. Errors in precise HR have been shown to result in chromosomal abnormalities, immunodeficiency, neurodegeneration and cancer susceptibility. Recruitment of *RAD51* to the sites for repair depends on proper functioning of the *RAD51* paralogs which include *RAD51B, RAD51C, RAD51D, XRCC1, XRCC2* and *XRCC3* [39, 40]. Mutations in these paralogs have been shown to specifically attenuate *RAD51* focus formation in response to irradiation and lead to the development of spontaneous chromosomal abnormalities [41]. Identification of multiple SNPs within this pathway may indicate a particularly important contribution of this pathway to long-term neurocognitive outcomes in this patient population. *RAD51, RAD52, BRCA1* and *BRCA2* genes have also been described as RAD associated factors important in RAD homologous recombination [41]. Furthermore, work by Berger et al., concluded that poor neurocognitive outcomes in children with pediatric brain tumors who underwent irradiation is likely associated with alterations in the RAD51 homologous recombination pathway [42].

While the non-dominant allele in *CYP2C9* was associated with a higher IQ in our results, the potential role of *CYP2C9* in cognition is still unknown at this time. One pilot program evaluating the use of pharmacogenetic-guided cannabis usage did find that there was low risk for patients with the dominant CYP2C9*1/*1 genotype having any acute negative cognitive or neurologic effects of the drug [43]. In patients with dementia and Alzheimer’s disease, *CYP2C9* is just one of several genes where the geno-phenotypes are involved in drug metabolism across several classes of drugs currently used to improve overall neurocognitive function [44, 45]. Further investigation of this gene and its relationship to neurocognitive outcomes is warrented.

Early recognition of patients at highest risk for cognitive deficits due to therapy is important on many levels. It may be possible to identify patients who should be considered for reductions in certain therapies to reduce the risk of neurocognitive side effects, without reducing their long-term survival rates. For example, the Children’s Oncology Group standard risk medulloblastoma trial (COG ACNS0331, NCI clinical trials identifier NCT00085735) studied the reduction of radiation to the craniospinal axis to determine if radiation can be safely dose reduced to minimize neurocognitive late effects while maintaining high survival rates. Unfortunately, this study found that a reduced CSI dose led to an unacceptable increase in event rates and decreased survival, although it was possible to utilize smaller boost volumes in the posterior fossa [46]. If children at high risk of cognitive deficits but low risk of relapse could be identified at diagnosis, these children could potentially be preferentially included in dose-reduced treatment arms.

For children where reduction of radiation therapy would result in an unacceptable increase in treatment failure, early cognitive intervention may be important. Research has demonstrated that education interventions may help with improving long-term fluid intelligence and therefore overall cognitive performance. One study found that a short, computerized training session with 4-year-old subjects was able to improve their working memory, indicating early intervention might ameliorate school delays [47]. Another study evaluated the use of a computer training program to improve the working memory and reduce learning deficits of children born at extremely low birth weight (ELBW). Former ELBW adolescents who underwent a 5-week intervention program improved both trained and non-trained working memory. More importantly, those with an IQ < 80 showed significant benefit, which was stable for at least 6 months after the training periods ended [48]. Cognitive rehabilitation has already been shown to improve fatigue, independence in activities of daily living, and overall cognitive function in pediatric cancer patients on therapy [49]. The Brainfit study is ongoing and looking specifically at cognitive and physical training as a potential treatment to improve neurocognitive outcomes in pediatric cancer survivors [37]. These studies highlight the importance of identifying children at risk for adverse neurocognitive outcomes, as there may be successful treatment strategies and potential interventions available to improve long-term educational and cognitive functioning.

We are not aware of any previous studies assessing genetic polymorphisms in DNA damage repair genes in pediatric brain tumor survivors. Strengths of our study included the patients in the two IQ groups were well matched in clinical demographics allowing for a more robust analysis of the selected genetic markers. Furthermore, even after adjusting for potential confounders, several SNPs showed significant differences in IQ changes. We recognize the limitation of a sample size of 46 subjects. The smaller number of patients resulted in insufficient power to include other recognized risk factors as co-variables in the analysis of SNPS association with neurocognitive performance.

Overall, these results support the hypothesis that somatic genetic variants in DNA repair genes may correlate with lower IQ scores in children treated for brain tumors and necessitate validation for potential use in clinical care and treatment planning. Development of a more robust sample population would allow for additional analysis including a GWAS adjusted for a wider selection of variables. Important future studies would include prospectively identifying patients with an at-risk SNP profile and evaluating what early interventions may support their overall neurocognitive outcomes.

### Supplementary Information

Below is the link to the electronic supplementary material.

Supplementary file1 (XLSX 19 KB)

## References

[1] National Brain Tumor Society Brain Tumor Facts. https://braintumor.org/brain-tumors/about-brain-tumors/brain-tumor-facts/. Accessed 26 Sept 2023

[2] Jemal A, Siegel R, Ward E, Hao Y, Xu J, Thun MJ (2009). Cancer statistics, 2009. CA Cancer J Clin.

[3] Institute NC (2020) Surveillance, Epidemiology and End Results Program. https://seer.cancer.gov/statfacts/html/childbrain.html

[4] Mulhern RK, Kepner JL, Thomas PR, Armstrong FD, Friedman HS, Kun LE (1998). Neuropsychologic functioning of survivors of childhood medulloblastoma randomized to receive conventional or reduced-dose craniospinal irradiation: a Pediatric Oncology Group study. J Clin Oncol.

[5] Lassaletta A, Morales JS, Valenzuela PL, Esteso B, Kahalley LS, Mabbott DJ, Unnikrishnan S, Panizo E, Calvo F (2023). Neurocognitive outcomes in pediatric brain tumors after treatment with proton versus photon radiation: a systematic review and meta-analysis. World J Pediatr.

[6] Carpentieri SC, Waber DP, Pomeroy SL, Scott RM, Goumnerova LC, Kieran MW, Billett AL, Tarbell NJ (2003) Neuropsychological functioning after surgery in children treated for brain tumor. Neurosurgery 52: 1348–1356 (**discussion 1356–1347**) 10.1227/01.neu.0000064804.00766.62 12762880

[7] Waber DP, Gioia G, Paccia J, Sherman B, Dinklage D, Sollee N, Urion DK, Tarbell NJ, Sallan SE (1990). Sex differences in cognitive processing in children treated with CNS prophylaxis for acute lymphoblastic leukemia. J Pediatr Psychol.

[8] Bledsoe JC, Breiger D, Breiger M, Shonka S, Ermoian RP, Ojemann JG, Werny DM, Leary SES, Geyer JR (2019). Differential trajectories of neurocognitive functioning in females versus males following treatment for pediatric brain tumors. Neuro Oncol.

[9] Palmer SL, Goloubeva O, Reddick WE, Glass JO, Gajjar A, Kun L, Merchant TE, Mulhern RK (2001). Patterns of intellectual development among survivors of pediatric medulloblastoma: a longitudinal analysis. J Clin Oncol.

[10] Jannoun L, Bloom HJ (1990). Long-term psychological effects in children treated for intracranial tumors. Int J Radiat Oncol Biol Phys.

[11] Rosser TL, Packer RJ (2003). Neurocognitive dysfunction in children with neurofibromatosis type 1. Curr Neurol Neurosci Rep.

[12] Chang L, Patel PP, Zhang Y, Cohen A, Cohen K, Jacobson L, Ladra M, Peterson RK, Acharya S (2023). Impact of socioeconomic status and chemotherapy on neurocognitive performance in children with brain tumors. Neuro-Oncol Pract.

[13] Demir-Lira OE, Prado J, Booth JR (2021). Neurocognitive basis of deductive reasoning in children varies with parental education. Hum Brain Mapp.

[14] Jalal S, Earley JN, Turchi JJ (2011). DNA repair: from genome maintenance to biomarker and therapeutic target. Clin Cancer Res.

[15] Hollegaard MV, Grauholm J, Borglum A, Nyegaard M, Norgaard-Pedersen B, Orntoft T, Mortensen PB, Wiuf C, Mors O, Didriksen M, Thorsen P, Hougaard DM (2009). Genome-wide scans using archived neonatal dried blood spot samples. BMC Genomics.

[16] Alan S, Kaufman SER, Coalson DL (2016). Intelligent testing with the WISC-V.

[17] Andreassen CN, Alsner J (2009). Genetic variants and normal tissue toxicity after radiotherapy: a systematic review. Radiother Oncol.

[18] Correa DD, Satagopan J, Cheung K, Arora AK, Kryza-Lacombe M, Xu Y, Karimi S, Lyo J, DeAngelis LM, Orlow I (2016). COMT, BDNF, and DTNBP1 polymorphisms and cognitive functions in patients with brain tumors. Neuro Oncol.

[19] Bunevicius A, Laws ER, Saudargiene A, Tamasauskas A, Iervasi G, Deltuva V, Smith TR, Bunevicius R (2019). Common genetic variations of deiodinase genes and prognosis of brain tumor patients. Endocrine.

[20] Correa DD, Satagopan J, Martin A, Braun E, Kryza-Lacombe M, Cheung K, Sharma A, Dimitriadoy S, O'Connell K, Leong S, Karimi S, Lyo J, DeAngelis LM, Orlow I (2019). Genetic variants and cognitive functions in patients with brain tumors. Neuro Oncol.

[21] Sepe DM, McWilliams T, Chen J, Kershenbaum A, Zhao H, La M, Devidas M, Lange B, Rebbeck TR, Aplenc R (2012). Germline genetic variation and treatment response on CCG-1891. Pediatr Blood Cancer.

[22] Kamdar KY, Krull KR, El-Zein RA, Brouwers P, Potter BS, Harris LL, Holm S, Dreyer Z, Scaglia F, Etzel CJ, Bondy M, Okcu MF (2011). Folate pathway polymorphisms predict deficits in attention and processing speed after childhood leukemia therapy. Pediatr Blood Cancer.

[23] Krull KR, Brouwers P, Jain N, Zhang L, Bomgaars L, Dreyer Z, Mahoney D, Bottomley S, Okcu MF (2008). Folate pathway genetic polymorphisms are related to attention disorders in childhood leukemia survivors. J Pediatr.

[24] Ishimaru S, Yuza Y, Kaneko T, Urashima M (2017). Effect of UGT2B17 deletion polymorphism on prognosis in pediatric cancer. Pediatr Int.

[25] Thiesen S, Yin P, Jorgensen AL, Zhang JE, Manzo V, McEvoy L, Barton C, Picton S, Bailey S, Brock P, Vyas H, Walker D, Makin G, Bandi S, Pizer B, Hawcutt DB, Pirmohamed M (2017). TPMT, COMT and ACYP2 genetic variants in paediatric cancer patients with cisplatin-induced ototoxicity. Pharmacogenet Genomics.

[26] Brown AL, Lupo PJ, Okcu MF, Lau CC, Rednam S, Scheurer ME (2015). SOD2 genetic variant associated with treatment-related ototoxicity in cisplatin-treated pediatric medulloblastoma. Cancer Med.

[27] Abo-Bakr A, Mossallam G, El Azhary N, Hafez H, Badawy R (2017). Impact of CYP1A1, GSTP1 and XRCC1 genes polymorphisms on toxicity and response to chemotherapy in childhood acute lymphoblastic leukemia. J Egypt Natl Canc Inst.

[28] Barahmani N, Carpentieri S, Li XN, Wang T, Cao Y, Howe L, Kilburn L, Chintagumpala M, Lau C, Okcu MF (2009). Glutathione S-transferase M1 and T1 polymorphisms may predict adverse effects after therapy in children with medulloblastoma. Neuro Oncol.

[29] Rednam S, Scheurer ME, Adesina A, Lau CC, Okcu MF (2013). Glutathione S-transferase P1 single nucleotide polymorphism predicts permanent ototoxicity in children with medulloblastoma. Pediatr Blood Cancer.

[30] Brackett J, Krull KR, Scheurer ME, Liu W, Srivastava DK, Stovall M, Merchant TE, Packer RJ, Robison LL, Okcu MF (2012). Antioxidant enzyme polymorphisms and neuropsychological outcomes in medulloblastoma survivors: a report from the Childhood Cancer Survivor Study. Neuro Oncol.

[31] Howarth RA, Adamson AM, Ashford JM, Merchant TE, Ogg RJ, Schulenberg SE, Ogg S, Li J, Wu S, Xiong X, Conklin HM (2014). Investigating the relationship between COMT polymorphisms and working memory performance among childhood brain tumor survivors. Pediatr Blood Cancer.

[32] Yilmaz B, Tokuc GA, Koc A, Yesil E (2017). Investigation of vitamin D receptor gene polymorphism in pediatric patients with brain cancer. Indian J Med Paediatr Oncol.

[33] Feldman D, Krishnan AV, Swami S, Giovannucci E, Feldman BJ (2014). The role of vitamin D in reducing cancer risk and progression. Nat Rev Cancer.

[34] Roth JJ, Santi M, Rorke-Adams LB, Harding BN, Busse TM, Tooke LS, Biegel JA (2014). Diagnostic application of high resolution single nucleotide polymorphism array analysis for children with brain tumors. Cancer Genet.

[35] Mascelli S, Nozza P, Jones DT, Colin C, Pistorio A, Milanaccio C, Ravegnani M, Consales A, Witt O, Morana G, Cama A, Capra V, Biassoni R, Pfister SM, Figarella-Branger D, Garre ML, Raso A (2016). TP53 codon 72 polymorphism may predict early tumour progression in paediatric pilocytic astrocytoma. Oncotarget.

[36] Brown AL, Sok P, Raghubar KP, Lupo PJ, Richard MA, Morrison AC, Yang JJ, Stewart CF, Okcu MF, Chintagumpala MM, Gajjar A, Kahalley LS, Conklin H, Scheurer ME (2023). Genetic susceptibility to cognitive decline following craniospinal irradiation for pediatric central nervous system tumors. Neuro Oncol.

[37] Benzing V, Eggenberger N, Spitzhuttl J, Siegwart V, Pastore-Wapp M, Kiefer C, Slavova N, Grotzer M, Heinks T, Schmidt M, Conzelmann A, Steinlin M, Everts R, Leibundgut K (2018). The Brainfit study: efficacy of cognitive training and exergaming in pediatric cancer survivors - a randomized controlled trial. BMC Cancer.

[38] Montour-Proulx I, Kuehn SM, Keene DL, Barrowman NJ, Hsu E, Matzinger MA, Dunlap H, Halton JM (2005). Cognitive changes in children treated for acute lymphoblastic leukemia with chemotherapy only according to the Pediatric Oncology Group 9605 protocol. J Child Neurol.

[39] Bonilla B, Hengel SR, Grundy MK, Bernstein KA (2020). RAD51 gene family structure and function. Annu Rev Genet.

[40] Takata M, Sasaki MS, Tachiiri S, Fukushima T, Sonoda E, Schild D, Thompson LH, Takeda S (2001). Chromosome instability and defective recombinational repair in knockout mutants of the five Rad51 paralogs. Mol Cell Biol.

[41] Wang J, Oh YT, Li Z, Dou J, Tang S, Wang X, Wang H, Takeda S, Wang Y (2021). RAD52 adjusts repair of single-strand breaks via reducing DNA-damage-promoted XRCC1/LIG3alpha co-localization. Cell Rep.

[42] Berger ND, Brownlee PM, Chen MJ, Morrison H, Osz K, Ploquin NP, Chan JA, Goodarzi AA (2022). High replication stress and limited Rad51-mediated DNA repair capacity, but not oxidative stress, underlie oligodendrocyte precursor cell radiosensitivity. NAR Cancer.

[43] Papastergiou J, Li W, Sterling C, van den Bemt B (2020). Pharmacogenetic-guided cannabis usage in the community pharmacy: evaluation of a pilot program. J Cannabis Res.

[44] Cacabelos R (2020). Pharmacogenomics of cognitive dysfunction and neuropsychiatric disorders in dementia. Int J Mol Sci.

[45] Cacabelos R, Naidoo V, Martinez-Iglesias O, Corzo L, Cacabelos N, Pego R, Carril JC (2022). Pharmacogenomics of Alzheimer's disease: novel strategies for drug utilization and development. Methods Mol Biol.

[46] Michalski JM, Janss AJ, Vezina LG, Smith KS, Billups CA, Burger PC, Embry LM, Cullen PL, Hardy KK, Pomeroy SL, Bass JK, Perkins SM, Merchant TE, Colte PD, Fitzgerald TJ, Booth TN, Cherlow JM, Muraszko KM, Hadley J, Kumar R, Han Y, Tarbell NJ, Fouladi M, Pollack IF, Packer RJ, Li Y, Gajjar A, Northcott PA (2021). Children's Oncology Group Phase III Trial of Reduced-Dose and Reduced-Volume Radiotherapy With Chemotherapy for Newly Diagnosed Average-Risk Medulloblastoma. J Clin Oncol.

[47] Bergman Nutley S, Soderqvist S, Bryde S, Thorell LB, Humphreys K, Klingberg T (2011). Gains in fluid intelligence after training non-verbal reasoning in 4-year-old children: a controlled, randomized study. Dev Sci.

[48] Lohaugen GC, Antonsen I, Haberg A, Gramstad A, Vik T, Brubakk AM, Skranes J (2011). Computerized working memory training improves function in adolescents born at extremely low birth weight. J Pediatr.

[49] Akel BS, Sahin S, Huri M, Akyuz C (2019). Cognitive rehabilitation is advantageous in terms of fatigue and independence in pediatric cancer treatment: a randomized-controlled study. Int J Rehabil Res.

